# Parking behaviour under the influence of alcohol

**DOI:** 10.1007/s00414-023-03082-2

**Published:** 2023-09-22

**Authors:** A. Tank, T. Tietz, J. Loskant, K. Zube, S. Ritz-Timme, B. Hartung

**Affiliations:** 1 Institute of Legal Medicine, University Hospital Duesseldorf, Duesseldorf, Germany; 2 Labor Lademannbogen MVZ GmbH, Hamburg, Germany; 3 Institute of Mathematics, Heinrich Heine University Duesseldorf, Duesseldorf, Germany; 4 DEKRA Automobil GmbH Duesseldorf, Duesseldorf, Germany; 5 Institut of Legal Medicine, University Hospital Essen, Essen, Germany

**Keywords:** Alcohol, Alcohol intoxication, Parking accidents, Driving under the influence, Driving impairment, Legal prosecution

## Abstract

Real-life driving studies evaluating the impact of alcohol influence on the ability to park a car are rare but necessary to assess a possible impairment to drive a car in the event of prosecution. In this study, 29 test persons (13 m, 16 f) completed three test drives with real cars, each made up of three different parking situations. While four test persons remained sober, the majority drank a previously calculated amount of alcohol before the second drive; the aim was to reach a blood alcohol concentration (BAC) of 1.1 g/kg. The third drive took place about 2 h later without any further ingestion of alcohol. The impact of BAC on the number of accidents, time needed to finish the drive, the amount of correction moves and quality of the final parking position (in the centre of the parking space) were analysed. Furthermore, pressure measuring films were applied to the test cars, measuring the average pressure and load in the areas of the accident impact. A significant increase of accidents could be noted with rising BAC. While a single accident happened to both sober and drivers under the influence of alcohol, more than one accident was only seen in drivers after the ingestion of alcohol (> 0.63 g/kg). The BAC had no impact on the other considered aspects. Concludingly, more than one impact site or accident while parking a car can serve as an indication for alcohol impairment of the driver at the time of the accident.

## Background

Driving under the influence of alcohol is a severe risk factor for causing an accident, particularly with higher blood alcohol concentrations (BAC) [1, 2]. Findings from the DRUID project in 2013 conclude that even BAC below 0.5 g/kg are concomitant with an elevated relative risk of being injured or killed in an accident: between 0.5 and 0.8 g/kg, the relative risk is 2–10 [1]. Compton et al. concluded that drivers with a BAC of 0.5 g/kg are 2.05 times more likely to crash than sober drivers [2]. In Germany, driving under the influence of alcohol can be punished as an administrative, as well as a criminal offence, depending on the circumstances and the BAC of the driver: whereas the determination of a BAC **≥** 1.10 g/kg will always be judged as a criminal offence, a BAC between 0.30 and 1.09 g/kg requires proof that the intoxication of alcohol actually had an impact on the fitness to drive in order to be prosecuted. Certain types of accidents can serve as such proof if they are typical for alcohol and far less common among sober subjects. Known studies regarding parking accidents are usually based on statistical and/or retrospective investigations, which bear the problem of drinking after driving, and/or a relevant delay between the accident and blood withdrawal with alcohol elimination. Some types of accidents, i.e. lane deviation accidents or driving straight ahead in a curve, have been proven to have a strong correlation to alcohol intoxication and are therefore suited as a proof for a reduced fitness to drive due to alcohol consumption. In other cases, a relevant correlation is harder to justify or can even be doubted, the latter including accidents occurring while parking or manoeuvring a car [3].

Actual real-life studies considering differing parking situations at different BAC are required to answer the question if alcohol consumption results in a significantly elevated risk for parking accidents; however, such studies are extremely rare. To the authors’ knowledge, the existing studies are not suitable to satisfyingly answer this question, as their primary focus lay on other topics and dedicated evaluations of parking abnormalities and mistakes were not executed [4, 5]. This study aims to contribute to this problem by providing prospective data.

Additionally, the question of noticeability of the occurring accidents had been addressed as well, since in court, the accused persons and their lawyers often confront the consulted experts with the statement that the accident had not been seen, heard or felt. Several studies have addressed this matter [e.g. 6-9] and concluded that the assessment of whether an accident could or rather should have been noted is complex: the optical noticeability can be affected by line-of-sight obstructions, the acoustical noticeability requires the sound of the accident differing distinguishably from the ambient noise, and the tactile noticeability ultimately depends on the materials affected and therefore the site of impact (i.e. softer doors or more rigid bumpers). Regarding the number of variables needed to be considered to answer this question to its full extent, this matter did not form the main focus of our study [10].

## Method

Driving experiments were conducted using real cars. Twenty-nine test persons participated (13 m, 16 f). The test persons were healthy, on average 31 years old (range: 22–57 years; median:27 years) and in possession of a valid driving licence for 14 years on average (range: 5–39 years; median: 10 years).

**Fig. 1:**
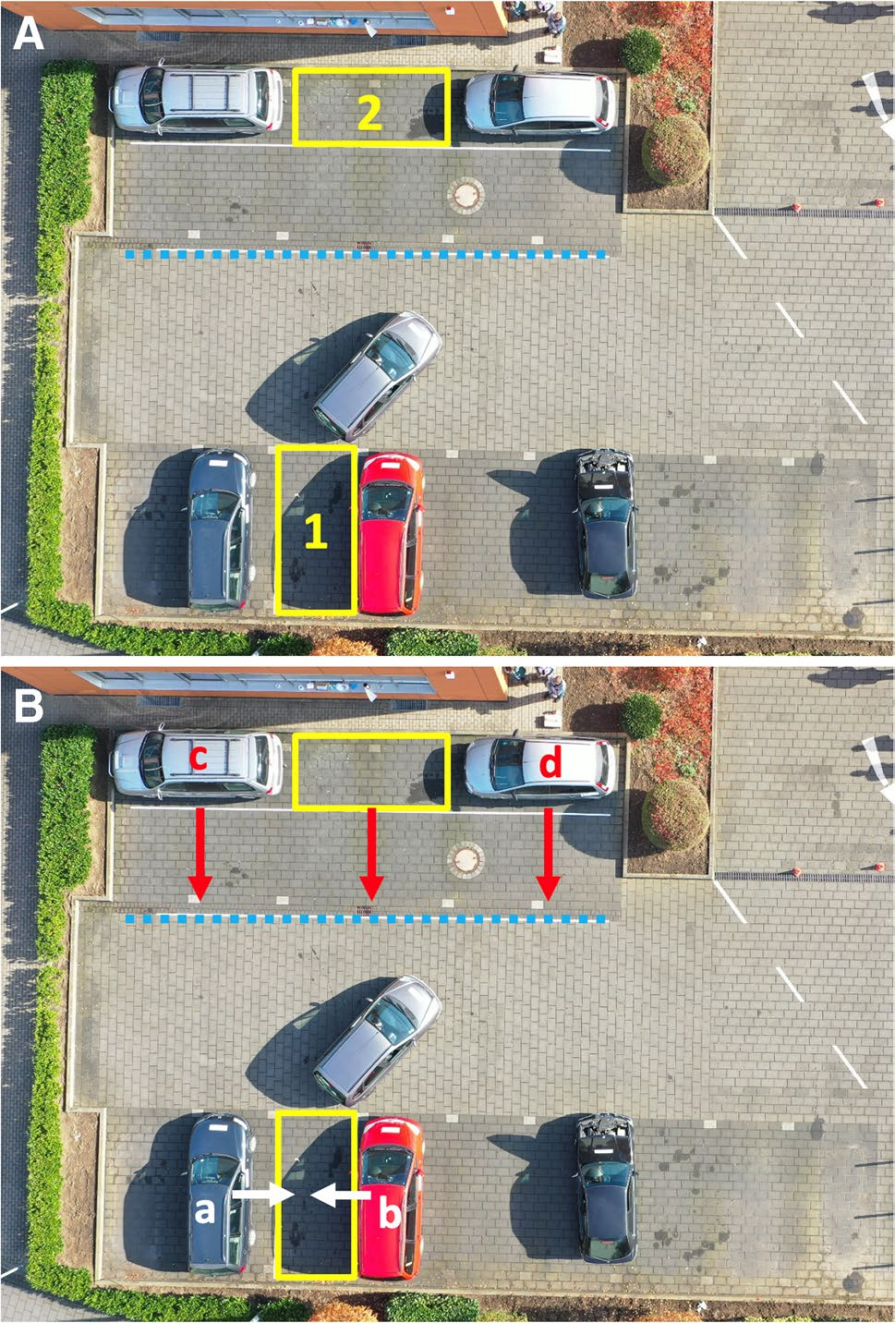
**a** Test area (day 1): the drivers had to complete three parking manoeuvres: forward (manoeuvre 1) and backward (manoeuvre 2) into parking space “1” measuring 2.25 × 5.00 m; afterwards, sideways backwards into parking space “2” measuring 2.20 × 5.25 m (manoeuvre 3). While turning the car between the forward and backward manoeuvre, the driven car was not allowed to cross the dotted blue line. **b** On the second day, cars a and b were parked slightly closer to each other without affecting the white landmarks of the parking space: 2.65 m versus 3.25 m the previous day. Cars c and d, together with the parking space created in between, were moved forward without affecting the space provided for turning the car. The kerb was then simulated using heavy wooden euro pallets

The test persons completed three test drives that were each composed of three different parking situations (forward and backward in one parking space typically found in parking lots as well as sideways in another parking space typically found at roadsides) (Fig. 1a). The experiments were conducted on 2 days; at each test day, they lasted from 10 AM to approximately 7 PM. Due to external circumstances, the experimental set-up differed slightly on the second day (Fig. 1b). However, the size of the actual parking spaces as well as the space given to turn the car in between those manoeuvres was not affected by this.

The test area with the parking lots used for testing was on private ground; the access could be closed by barriers. A sober co-driver was present in each of the two test cars at each drive. As two different models were used as test cars, the length of the cars varied slightly: the first car was a Renault Megane Scenic (4.1 × 1.7 m) and the second a BMW 3 compact (4.2 × 1.7 m). Both cars were restricted to a maximum speed of approximately 20 km/h using a mechanical restriction. The cars did not feature parking aids of any kind and offered a manual transmission system. All test subjects used the same car for each of their drives after they had customized to the course.

Four of these test persons (2 m, 2 f) stayed sober throughout the test drives to assess possible changes caused by tiredness or further habituation to the cars as possible confounders to the test results. Alcohol-consuming test persons drank a previously defined amount of alcohol after habituation to the course and the first drive (V0: sober drive), which served as the baseline. Within 2 h, the achievement of a maximum blood alcohol concentration (BAC) of 1.10 g/kg was intended, calculated by the formula of Widmark [11]. Right after the consumption of the precalculated amount of alcohol, the second drive took place in the phase of maximum BAC (V1). The third drive took place another 2 h later in the phase of alcohol resorption (V2). After each drive, blood samples were taken.

All drives were videotaped, and the type and number of accidents, time to finish the parking situations, amount of correction moves and quality of the final parking position (in the centre of the parking space, allowing all passengers to exit the car evenly well) were analysed.

**Fig. 2:**
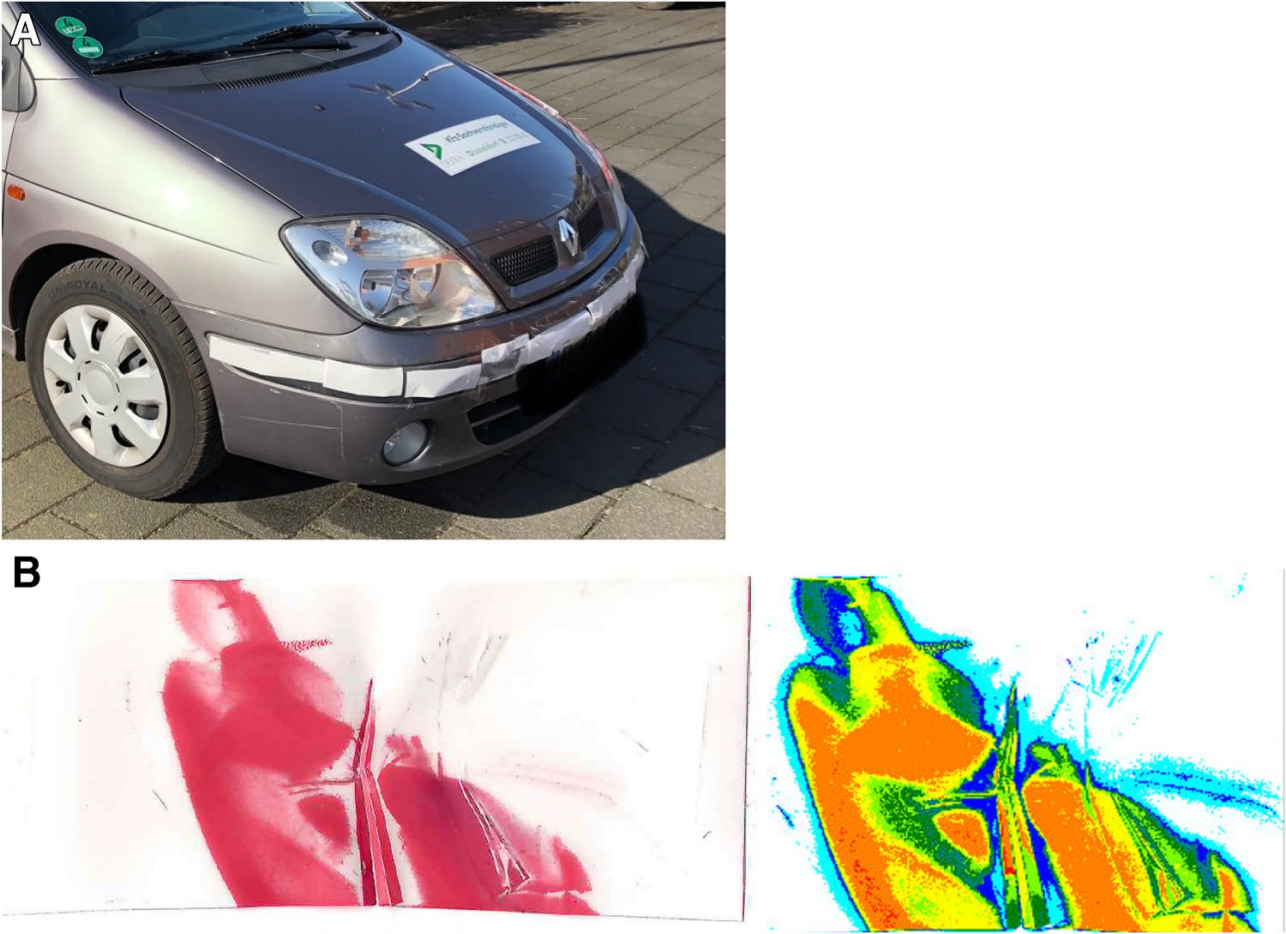
**a** Example of the film being applied to the front bumper of one of the test cars. **b** Fujifilm prescale (MS) pressure film after impact (left) and after digital evaluation (right)

In order to further objectify the accidents, pressure measurement films (Fujifilm Prescale) that were successfully tested when measuring blunt force [12] were attached to the test cars at locations typical for impacts (in particular the bumpers; Fig. 2a). The film allowed a digital analysis of the size of the impacted area, the average pressure in those areas and the product of both, the so-called load (Fig. 2b).

The drivers as well as the co-drivers were asked whether accidents have been noticed and, if so, in what way (visual, acoustical or tactile perception).

### Statistical analysis

Multiple statistical analyses were performed, aiming to analyse the impact of BAC on driving performance.

In all analyses, a mixed linear model was used accounting for individual differences between drivers. All models included the BAC as explanatory variable as well as different individuals driving the car as random effects. To account for a possible learning effect, the drive number (V0, V1, V2) was included as further explanatory variable, thereby enabling the model to attribute the possible learning effect to this variable and not to the BAC. The learning effect is expected to be small, as every test person was given time to properly adapt to the cars and course prior to the first test drive.

In order to measure driving performance, four different target variables were considered in four separate analyses:

- The number of accidents
- The number of corrections made until reaching the final parking position
- The quality of the final parking position (good/not good in terms of a central position in the given parking space)
- The duration (in seconds) needed to reach the final parking position

Furthermore, the intensity and noticeability of the accidents were analysed descriptively, without employing any statistical tests, as the number of samples was too small for a valid statistical analysis.

## Results

The test persons completed an overall amount of 87 test drives (29 drivers with 3 drives each), no driver dropped out and no drive was aborted.

In 20 of these 87 drives, at least one accident occurred. Most of the accidents happened while parking the car at the roadside (sideways resp. manoeuvre 3), where in 17 drives, one or more accidents occurred. The other accidents happened while parking the car forward (*N*=1; manoeuvre 1) and backward (*N*=1; manoeuver2) and while turning in between the parking manoeuvres (*N*=2). The most common impact sites were the central front (*N*=14) and central back bumpers (*N*=7).

During the first (sober) drive, all test persons had a BAC of 0.0 g/kg. The analysis of BAC in the second and third drive in the state of alcoholization gave the results seen in Table 1.

**Table 1:** BACs of the alcohol-consuming test persons in the second and third test drive

Drive	Average	Median	Lowest BAC	Highest BAC
Second (V1)	0.99 k/kg	0.97 k/kg	0.63 g/kg	1.40 g/kg
Third (V2)	0.74 g/kg	0.75 g/kg	0.37 g/kg	1.36 g/kg

Accidents were noted with influenced drivers as well as with sober ones: 7 drivers caused at least one accident in their sober drive (V0), 7 immediately after consuming alcohol (with maximum BAC, V1) and 6 in their last drive (V2).

Five test drives exhibited more than one accident that almost all occurred during parking manoeuvre 3. Only in one of these cases an accident occurred while turning the car, followed by a second accident during manoeuvre 3.

### Correlation between BAC and the number of accidents

**Fig. 3:**
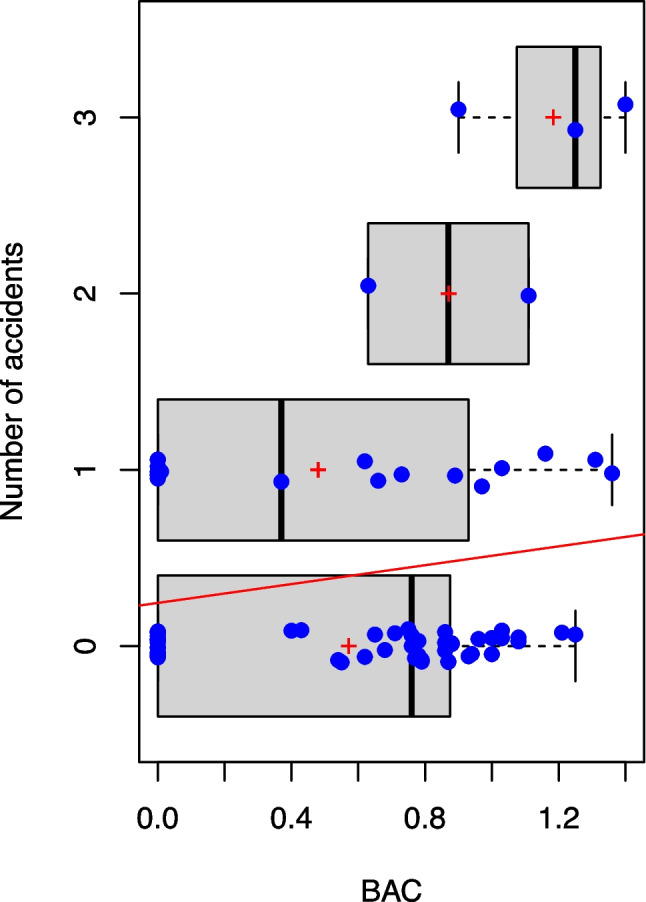
Number of accidents in relation to the BAC. The blue dots represent the single values of the test persons over all three test points. 50 % of all values are within the box, the 25 % highest, and lowest values are marked by whiskers. The black lines visualize the median; the red crosses the average. The red line depicts the line of regression

Overall, a significant rise of accidents was seen with increasing BAC (*p=0.04*), as depicted in Fig. 3 with the red line. Especially more than one accident correlated with a significantly higher BAC, as they only occurred in alcoholized drivers (minimum BAC: 0.63 g/kg). The occurrence of one accident however, was seen in sober and influenced drivers alike, with no significant difference (Fig. 3).

### The correlation of BAC and the quality of parking

The quality of parking, measured by the amount of required correction moves and the final parking position in the centre of the parking space, was not significantly affected by the BAC (*p*=0.25 and *p*=0.75). The average amount of corrections needed to park the car throughout all three drives were 1 (manoeuvre 1), 1 (manoeuvre 2) and 2 (manoeuvre 3).

### The correlation of BAC and the time to finish the drive

**Fig. 4:**
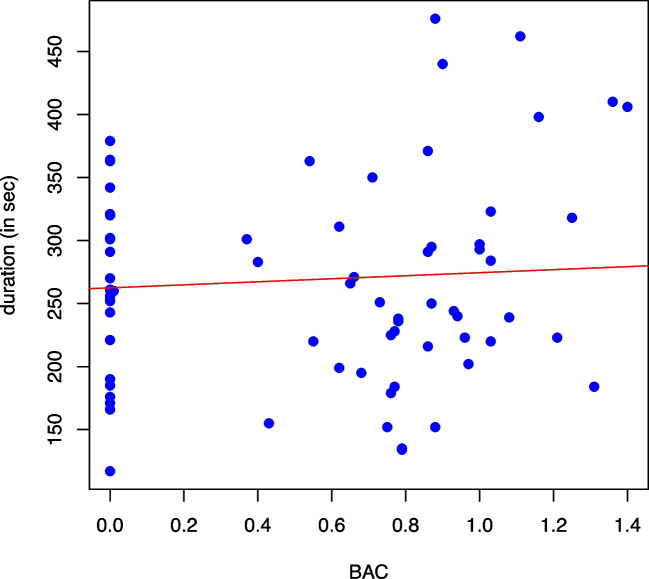
Time needed to finish the drive in seconds in relation to the BAC. Blue dots: single values of the test persons; red line: regression line

In our study, higher BACs had no significant effect on the time to finish the test course (*p*=0.41; Fig. 4).

### The influence of BAC to the intensity and noticeability of accidents

Since the overall number of accidents was comparably low and not all of them left visual marks on the applied film, a statistical evaluation of a possible correlation of the impacted pressure and BAC was not possible.

**Fig. 5:**
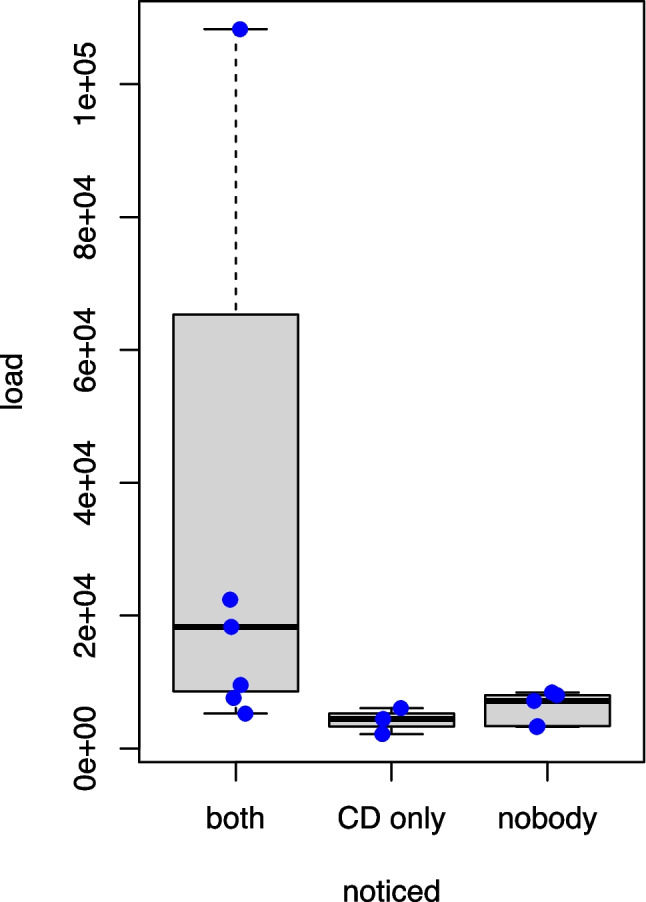
The load in relation to the noticeability: accidents noticed by both (*n*=7), by none (*n*=5) or the co-driver (CD) only (*n*=3). The blue dots represent the single values. 50 % of all values are within the box, the 25 % highest and lowest values are marked by whiskers. The black lines represent the median

Fifteen films allowed digital evaluation, 4 of these showed the result of two or three accidents with similar impact sides, as the film was not renewed after each accident but after each test drive. For the following analysis, these were treated as one accident since a differentiation was not possible. The average pressure of the determined accidents ranged from 9 to 27 MPa, the load from 2130 to 338694 N (Table 2, Fig. 5).

**Table 2:** Average pressure and load of the accidents noticed by both driver and co-driver, co-driver only and none of the passengers in comparison

Noticed by	*n*	Average pressure (MPa)	Load (N)	
		Range	Range	Mean
Both	7	9.8–27.0	5225–338694	72856
Co-driver only	3	9.8–18.3	2130–6034	4194
None	5	9.0–18.0	3204–8393	6010

Seven of these accidents were noted by driver and co-driver alike; they had all been tactilely noticed by both passengers. Five accidents were noticed by none of the passengers. Three were noticed only by the co-driver: in one case, the co-driver clearly saw the accident but neither heard nor felt it; the impact location was the front bumper’s right site. The other two accidents were heard by the co-driver, but neither seen nor felt. The drivers offered BAC of 0.0 g/kg, 0.63 g/kg and 1.11 g/kg at that time. Due to prevention strategies during the COVID-19 pandemic, the car windows were open during the drives.

## Discussion

More accidents were seen with increasing BAC. Yet, a parking accident cannot be interpreted as proof of a reduced fitness to drive due to alcohol in general. A singular accident (*N*=15) was not correlated to a higher BAC.

As a main result of this study, more than one accident (*N*=5) only occurred with a BAC >0.63 g/kg and therefore seems to be a suitable indicator for a reduced fitness to drive due to alcohol intoxication. Supporting this finding is the fact that these accidents either occurred within the same parking situation or while directing the car to this space, followed by another accident trying to park the car in it. Parallel parking at the roadside was involved in all these accidents. Besides some practice, this parking manoeuvre requires a good sense of distance estimation, which has been shown to be affected by alcohol by other authors [13, 14].

In our study, the BAC of the driver was not correlated to the intensity (average pressure and load at the impact site) of an occurring accident. The question of potential noticeability is a complex one and can only partially be addressed here. Every accident that both passengers noticed had also been tactilely felt by both. The ones that were only noted by the co-driver, however, were tactilely felt by none of them but only heard or seen by the co-driver. While the tactile component in our study was felt throughout the car, the acoustic and visual component seemed to depend more on the person’s location within the car. The BAC did not have a significant influence on whether the driver noticed the accident.

In court, when trying to assess the driver’s fitness under the influence of alcohol after a parking accident, the circumstances have to be enquired as detailed as possible. According to this study’s results and under the precondition that other distinctive features of the driver can be negated, only causing more than one accident while parking a car justifies the assumption of an alcohol-related impairment of the driver at that time and insofar the pursuit as a criminal offence.

It can be concluded that two or more accidents in isolated parking situations do not regularly occur if the driver is sober. Parking at the side of the road in our study turned out to be particularly challenging. This is crucial for the assumption of relative driving impairment according to the German criminal code (§316 StGB). Therefore, special attention should be turned to witness statements and the existence of multiple impact sites of the car/cars involved in the accident.

## Limitations

The study was subject to different limitations.

- First, a small collective of test persons was included and the control group consisted of four persons only.
- Secondly, all test subjects were fully aware of the test situation and highly motivated to perform as well as possible.
- Thirdly, a learning effect throughout the study was noted but could be considered when statistically analysing the results.
